# Tracking discussions of complementary, alternative, and integrative medicine in the context of the COVID-19 pandemic: a month-by-month sentiment analysis of Twitter data

**DOI:** 10.1186/s12906-022-03586-1

**Published:** 2022-04-13

**Authors:** Jeremy Y. Ng, Wael Abdelkader, Cynthia Lokker

**Affiliations:** Department of Health Research Methods, Evidence, and Impact, Faculty of Health Sciences, McMaster University, Hamilton, ON Canada

**Keywords:** Complementary and alternative medicine, COVID-19, Sentiment analysis, Twitter, Social media

## Abstract

**Background:**

Coronavirus disease 2019 (COVID-19) is a novel infectious disease caused by the severe acute respiratory syndrome coronavirus 2 (SARS-CoV-2). Despite the paucity of evidence, various complementary, alternative and integrative medicines (CAIMs) have been being touted as both preventative and curative. We conducted sentiment and emotion analysis with the intent of understanding CAIM content related to COVID-19 being generated on Twitter across 9 months.

**Methods:**

Tweets relating to CAIM and COVID-19 were extracted from the George Washington University Libraries Dataverse Coronavirus tweets dataset from March 03 to November 30, 2020. We trained and tested a machine learning classifier using a large, pre-labelled Twitter dataset, which was applied to predict the sentiment of each CAIM-related tweet, and we used a natural language processing package to identify the emotions based on the words contained in the tweets.

**Results:**

Our dataset included 28 713 English-language Tweets. The number of CAIM-related tweets during the study period peaked in May 2020, then dropped off sharply over the subsequent three months; the fewest CAIM-related tweets were collected during August 2020 and remained low for the remainder of the collection period. Most tweets (*n* = 15 612, 54%) were classified as positive, 31% were neutral (*n* = 8803) and 15% were classified as negative (*n* = 4298). The most frequent emotions expressed across tweets were trust, followed by fear, while surprise and disgust were the least frequent. Though volume of tweets decreased over the 9 months of the study, the expressed sentiments and emotions remained constant.

**Conclusion:**

The results of this sentiment analysis enabled us to establish key CAIMs being discussed at the intersection of COVID-19 across a 9-month period on Twitter. Overall, the majority of our subset of tweets were positive, as were the emotions associated with the words found within them. This may be interpreted as public support for CAIM, however, further qualitative investigation is warranted. Such future directions may be used to combat misinformation and improve public health strategies surrounding the use of social media information.

## Background

Coronavirus disease 2019 (COVID-19) is a novel infectious disease caused by severe acute respiratory syndrome coronavirus 2 (SARS-CoV-2) [1]. In December 2019 it was first discovered, having originated from Wuhan, China, and has since rapidly spread across the globe, with 220 countries reporting cases. As of March 23, 2022, over 452.2 million cases and 6.03 million deaths have been reported by the World Health Organization (WHO) [2]. Common symptoms associated with COVID-19 include fever, tiredness, and dry cough, but can also include aches and pains, nasal congestion, runny nose, sore throat or diarrhea. While some patients infected with the disease do not exhibit symptoms, COVID-19 is of great concern to global public health as approximately 5% of people who are infected will become seriously ill and need intensive care [1]. Certain health precautions such as frequent and thorough hand washing, social distancing, wearing masks, and self-isolation have been shown to reduce the spread of COVID-19 [3]. There were no proven drugs to prevent or cure COVID-19 at the outset of the pandemic [4, 5], and vaccines became available in 2021 [6, 7]. Despite this, and even with the administrative of over 10.7 billion vaccine doses administered to date, some complementary, alternative, and integrative medicines (CAIMs) have been touted as the solution [8].

According to the National Center for Complementary and Integrative Health (NCCIH), complementary and alternative medicine is defined as “health care approaches that are not typically part of conventional medical care or that may have origins outside of usual Western practice”. “Complementary” refers to care in combination with conventional medicine, whereas “alternative” refers to care in place of it. “Integrative medicine” refers to bringing conventional and complementary approaches together in a coordinated way [9]. While the use of CAIMs in the context of some diseases have been shown to be effective or promising, it is also well-documented in the research literature that CAIM is sometimes promoted as a remedy for which the evidence-base is lacking [10, 11]. This is further compounded by the fact that many patients assume that CAIM is both safe and effective, even though both CAIM therapies and practitioners are generally subject to less regulation [12]. There is a growing movement of conventional and CAIM practitioners working together to support the safer and more effective uses of CAIM therapies, but concerns remain about misinformation circulated online [13–15]. Of particular interest is social media, as the body of literature that has considered its impact and growing significance as a source of health information for the general public has grown over recent years [16–18]. Emerging methodologies that have been employed to study social media content include the utilization of natural language processing (NLP), which is defined by Liddy [19] as “a theoretically motivated range of computational techniques for analyzing and representing naturally occurring texts at one or more levels of linguistic analysis for the purpose of achieving human-like language processing for a range of tasks or applications”. One of the subfields of NLP is sentiment analysis, which automatically classifies text according to the polarity (positive to negative) of the sentiments expressed therein [20]. A positive and negative sentiment can be defined as a favourable and unfavourable expression towards a subject, respectively, while a neutral sentiment represents an expression that is neither favourable nor unfavourable.

In the context of recently past pandemics, such as influenza-A (H1N1), NLP analyses of social media content (e.g. Twitter) served multiple purposes, including monitoring, predicting, and tracking levels of infection, and identifying the kinds of information circulated, distilled into content categories [21–24]. To our knowledge, a very limited amount of research has been conducted at the intersection of CAIM and social media [25, 26], while no studies have ever investigated what information surrounding CAIM is communicated across social media during any pandemics that have occurred since the inception of the Internet. In the present study, we conducted a sentiment analysis with the intent of understanding what kind of CAIM content related to COVID-19 is being generated on Twitter during the pandemic. We identified Twitter as our social media platform of choice since it is easy to use, cheap, and accessible, and the data can be easily collected in comparison to other platforms that have more restrictive privacy policies [20]. As the first study of its kind, our findings provide insight into a previously unexplored environment in the context of CAIM, that is both popular and free to patients, yet rife with quickly and continuously generated information of unassessed quality.

## Methods

### Approach

**Fig. 1:**
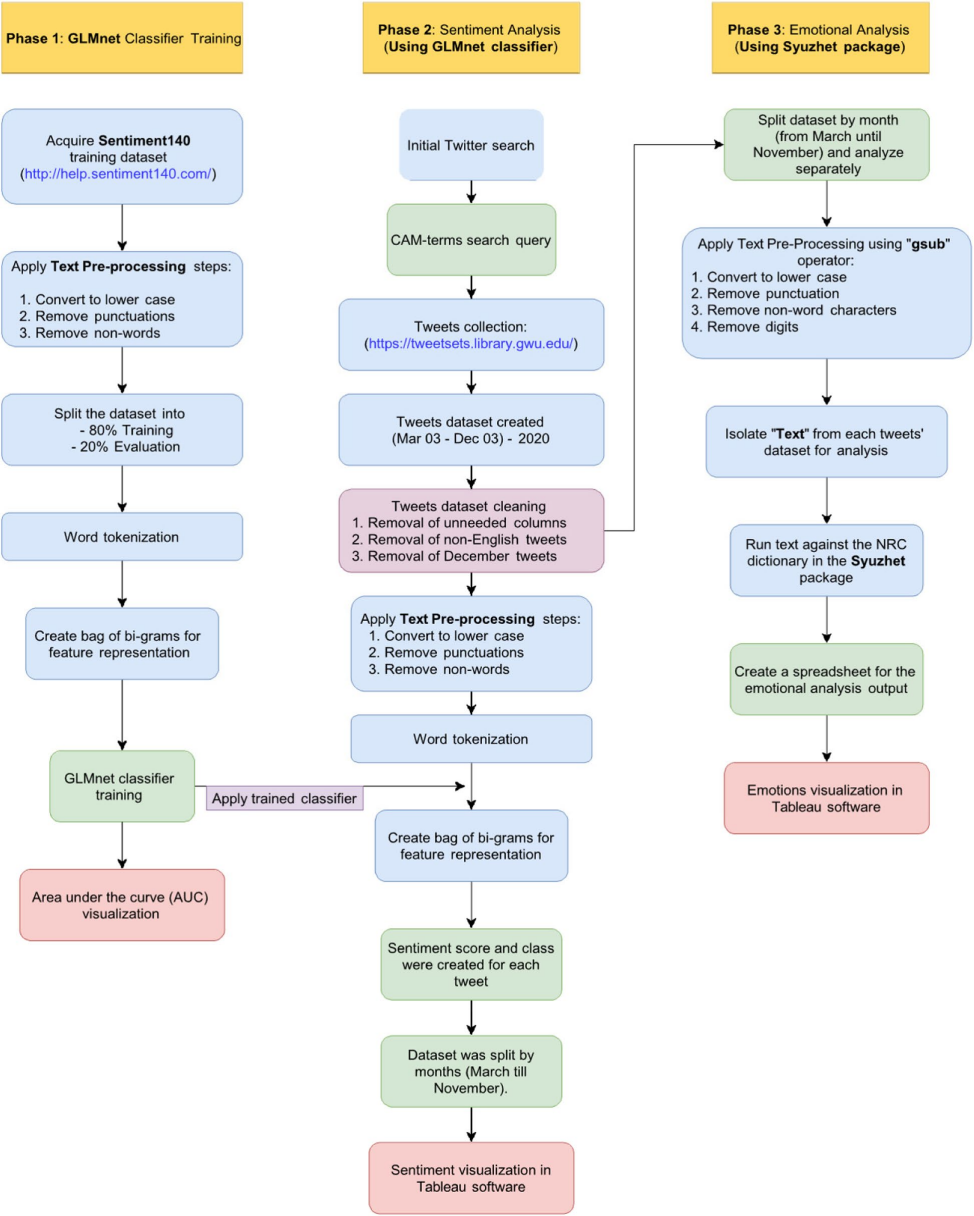
Flowchart depicting the steps taken for the sentiment and emotion analysis of CAIM-related COVID-19 tweets

We used a supervised machine learning approach, in which the machine algorithm is given labelled data—a dataset that has been classified—to be used for predicting the classification of the targeted unlabelled data, in our case CAIM-related tweets [27]. Overall, our approach consisted of the following 2 phases: 1a) training and testing a machine learning classifier using a large, pre-labelled Twitter dataset, 1b) using the trained classifier to predict the sentiment class of each tweet, and 2) utilizing an NLP package to identify the emotions based on the words contained in the tweets. We first searched for CAIM-related tweets from within a set of COVID-19-filtered tweet dataset using CAIM-related search terms. All tweets analysed in this study, therefore, contained at least one CAIM-related word/term and at least one COVID-19-related word/term. We then obtained the training dataset; a large dataset of tweets that have been pre-labelled based on positive and negative sentiments created by Go et al. [28] and made publicly available through the Sentiment140 website [29]. In short, a sentiment can be defined as a “positive or negative feeling”, and thus training data hand-labelled by humans can be subject to a great degree of subjectivity. We chose Sentiment140 as our training dataset which mitigates this to an extent, as the tweets in the dataset were machine-labelled based on the emoticons. For example, “:)” in a tweet indicates that the tweet contains positive sentiment and “:(“ indicates that the tweet contains negative sentiment. We used two supervised machine learning approaches to conduct both a sentiment analysis (using the GLMnet trained classifier [30]) and an emotion analysis (using Syuzhet NLP package in R [31]) of our CAIM-tweets dataset. Study steps are detailed in the following sections and depicted in a flowchart in Fig. 1.

### Development of search strategy

Preliminary searches of Twitter-related sentiment analyses yielded no consistent or standardized method for identifying search terms. In preparation for conducting searches across a large dataset of tweets, we first searched the Twitter platform using a number of CAIM-related and COVID-19-related terms to identify the most frequently used terms. Commonly used COVID-19-related terms were relatively simple to identify, as most Twitter users used the terms “COVID”, “coronavirus” or “COVID-19. Given the lack of consensus on a complete or comprehensive operational definition of CAIM [32], we browsed MeSH headings on MEDLINE and selected the most commonly used terms to refer to CAIM [33], and common CAIM systems and their respective practitioners (e.g., “homeopathy” vs. “homeopath”, etc.) [9].We excluded highly specific or specialized types of CAIM that would not typically be used by the general public (e.g., “electroacupuncture” as opposed to “acupuncture”, the specific genus and species of herbs as opposed to a generic term such as “herbal medicine”, etc.). A shortlist of 44 CAIM-related terms were combined with the 3 COVID-19-related terms, resulting in 132 unique Twitter searches. After applying these searches to Twitter, we looked at the recency of the use of terms to identify those most relevant to include in our final search strategy. Based on this approach, our final CAIM search strategy included the following terms: “Ayurveda”, “Ayurveda medicine”, “dietary supplement”, “herbal”, “herbal medicine”, “herbal supplement”, “herbal therapy”, “herbalism”, “herbs”, “homeopathy”, “homeopathic”, “natural medicine”, “natural medicines”, “natural therapies”, “natural therapy”, “naturopathic medicine”, “naturopathy”, “traditional medicine”, “traditional medicines”, “vitamins”, and “vitamin”.

### Data collection

To collect tweets at the intersection of COVID-19 and CAIM, we applied our CAIM search strategy to a COVID-19 filtered tweets dataset made available by the TweetSets website [34, 35]. TweetSets is an open-source online platform from the George Washington University (GWU) that archives Twitter datasets for research purposes. GWU Dataverse is part of the Harvard Dataverse, a free data repository open to all researchers from any discipline, both inside and outside of the Harvard community [36]. TweetSets allows users to select, generate, and download tweet IDs from publicly available filtered tweets datasets by allowing for querying on keywords, hashtags, mentions, users, embedded media, and type of tweet (original, retweet, quotes, or reply). Through TweetSets, we accessed the Coronavirus dataset, created by Kerchner and Wrubel [37], which contained 354 903 485 COVID-19 related tweets from March 03, 2020 and November 30, 2020 as of February 03, 2021. GWU compiled the tweets by applying the keywords #Coronavirus, #Coronaoutbreak, #COVID19 using the post statuses/filter method of the Twitter stream application programming interface (API). We applied our CAIM-related search strategy to filter the Coronavirus dataset, thus identifying tweets containing both CAIM and COVID-19-related content. We limited tweets to original English-language tweets that included one or more of the CAIM-related search terms.

The TweetSets output was a condensed series of tweet IDs relating to the identity of each included tweet. To extract the text of the tweet, date of posting, user account identifiers, and tweet metadata (i.e., location coordinates, hashtags, tweets URL, retweet status, and language code), a “Hydrator” software [38] was used. This software allowed us to extract the tweet details from the tweet IDs in our search results. The output dataset was a comma-separated values (.csv) file that was imported into Microsoft Excel for data cleaning and analysis, which is described in further detail below.

### Sentiment analysis of CAIM-related tweets

Contextual polarity sentiment analysis involves determining the polarity of the opinion resulting in an output of positive, neutral, and negative [39]. Sentiment analyses of the collected tweets was performed in Rstudio software. The contextual polarity sentiment analysis was conducted using the Text2Vec package [40] for text processing, an R package which provides a framework for text analysis and NLP, and the GLMnet package [39] for the machine learning classifier. We used a supervised machine learning approach whereby the learning capabilities of the model was determined by a labelled training dataset. For this training, we used the Sentiment140 tweets dataset [29], which is a labelled dataset of 1.6 million twitter messages created by Go et al. [28] using machine learning to classify tweets into positive and negative based on their sentiments. The training dataset, Sentiment140, contained the targeted correct attributes (sentiment) from which the learning machine algorithm found patterns that mapped the input data attributes to the target (sentiment e.g., positivity, neutrality, negativity). The machine learning model functions by analysing the input (our tweet dataset) based on knowledge acquired from the training set, and then returning a predicted value related to the sentiment of each identified CAIM-related tweet. The training dataset was split into training and evaluation in an 80:20 ratio. Words in the training dataset were tokenized using the itoken() function in Text2Vec Package, a process of reducing a text into phrases or words called tokens. The aim of this process is to identify meaningful words in a given sentence since textual data is a stream of characters [41]. Prior to the tokenization, we applied some text pre-processing procedures to the training and testing datasets: each word was converted to lowercase, and symbols, numbers, and non-words were removed.

N-grams was used as our feature selection (i.e., the process of selecting a subset of relevant features (words, variables, attributes, or predictors)) for use in model construction. N-grams is a space reduction method that selects a subset of the dataset to identify more relevant features from the pre-processed text to improve classification quality and reduce computational complexity. N-gram is the sequence of a given number of words (N), and it is a probability model to predict the most probable word that might follow a certain sequence while preserving the word locality information; we used bi-grams which is the sequence of two words [42, 43]. For the machine to understand the text within our dataset, the text had to be vectorized in a process called text vectorization; in other words, this process transformed text into an array of numbers (vectors) to make it understandable by the machine [44]. Vectorized bi-grams were organized in a document-term matrix (DTM) —a mathematical matrix that describes the frequency of terms in a collection of texts [45]. A machine learning classifier, the algorithm for prediction of the target class label, was fit to the created DTM for training. The classifier output was set to generate fitted probabilities values for each tweet, with a score ranging between 0 and 1 (0 tending towards the most negative, 1 tending towards the most positive, and values between 0.35 and 0.65 being considered neutral [46]). We selected the regularized generalized linear model, GLMnet, as our classifier; this is an extension of the generalized linear model with built-in variable selection making them helpful in real world datasets. To decrease bias in the results of the classifier, we have used the fivefold cross validation. To evaluate the performance of our machine learning model as applied to the evaluation dataset, we determined the receiver operator characteristic (ROC) curve and area under the ROC curve (AUC).

### Emotion analysis of CAIM-related tweets

To further identify the emotions relayed within our tweet dataset, we split the dataset by month (nine datasets). Analysis was performed using the Syuzhet R package, which is capable of extracting sentiment and sentiment-derived plot arcs from text using a variety of sentiment dictionaries within the package [31]. Syuzhet employs a lexicon dictionary of emotions based on the National Research Council Canada (NRC) Emotion Lexicon [47, 48]. This lexicon was created by manual annotation of a list of English words and their associations with eight basic emotions (anger, fear, anticipation, trust, surprise, sadness, joy, and disgust) and two sentiments (negative and positive) accomplished by crowdsourcing. Tableau Desktop (Professional Edition) was used for the visualization of the results in terms of frequencies, percentage, and changes over time for the eight emotions.

## Results

### Tweet dataset

**Fig. 2:**
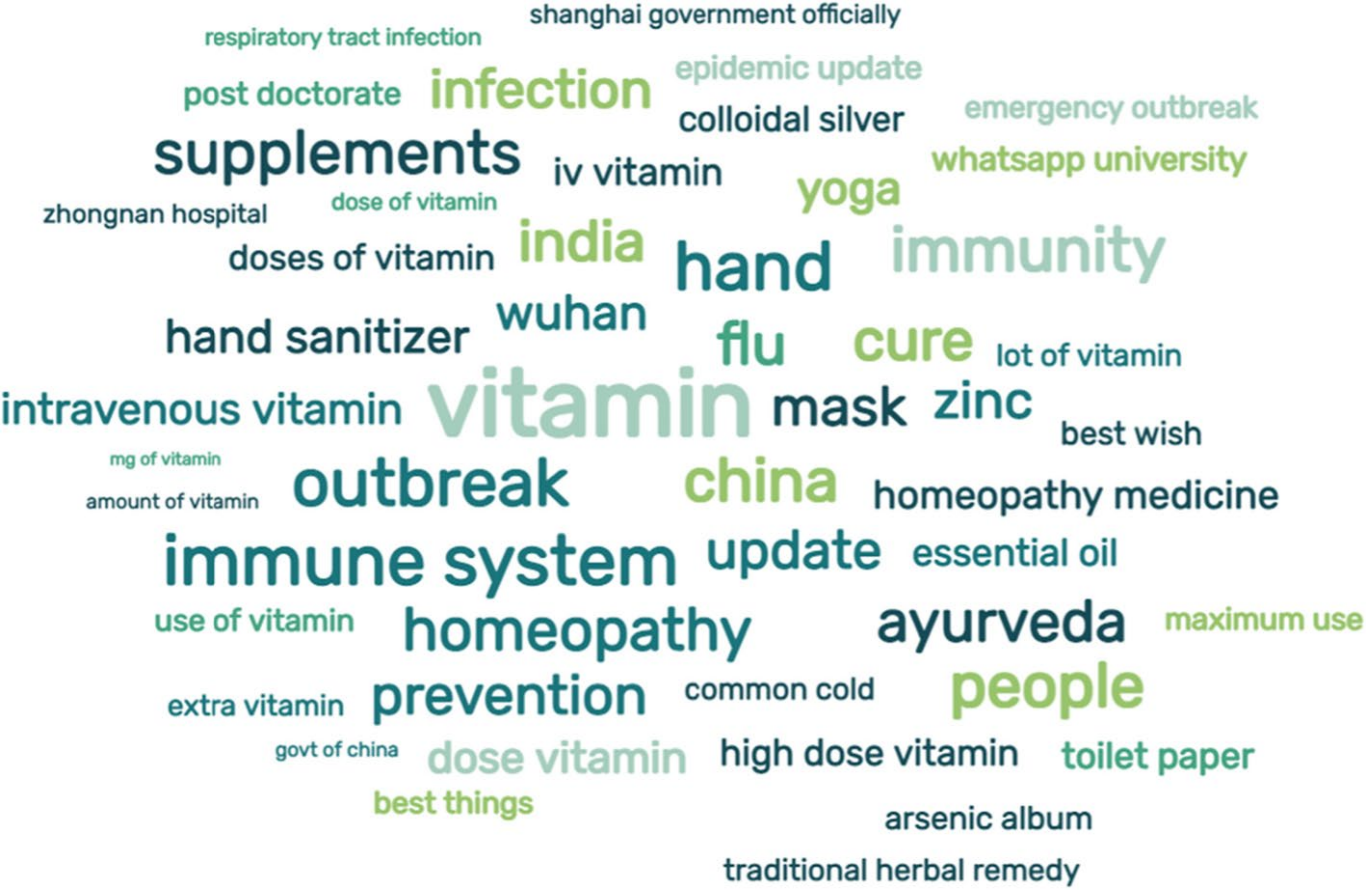
Word cloud depicting the most frequently mentioned words/terms contained in our subset of analyzed CAIM-related COVID-19 tweets

**Fig. 3:**
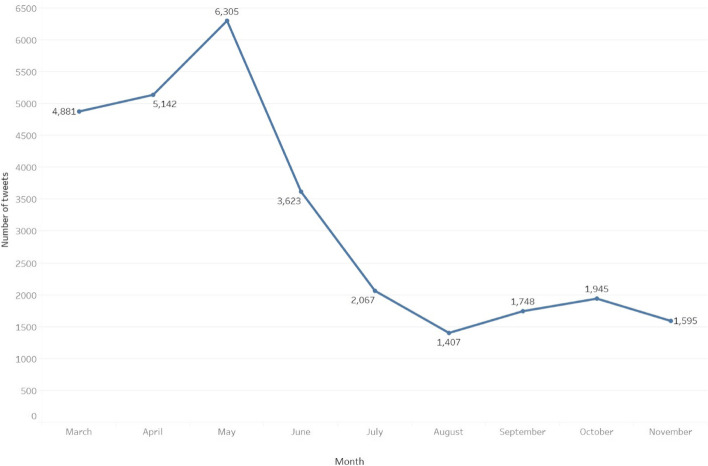
Frequency of CAIM-related tweets between March 03 and November 30, 2020 shown across monthly intervals

With our search terms, we identified 39 775 original tweets, of which 28 713 were posted in the English language. The most commonly used CAIM-related hashtags were #vitamin followed by #ayurveda. “Vitamin” and “vitamins” were overwhelmingly the most common CAIM-related terms followed by “herbal” and “Ayurveda”, as shown in Fig. 2. The number of CAIM-related tweets during our study period peaked in May 2020, then dropped off sharply over the subsequent three months; the fewest CAIM-related tweets were collected during August 2020 and remained low for the remainder of the collection period (Fig. 3).

### Sentiment analysis

**Fig. 4:**
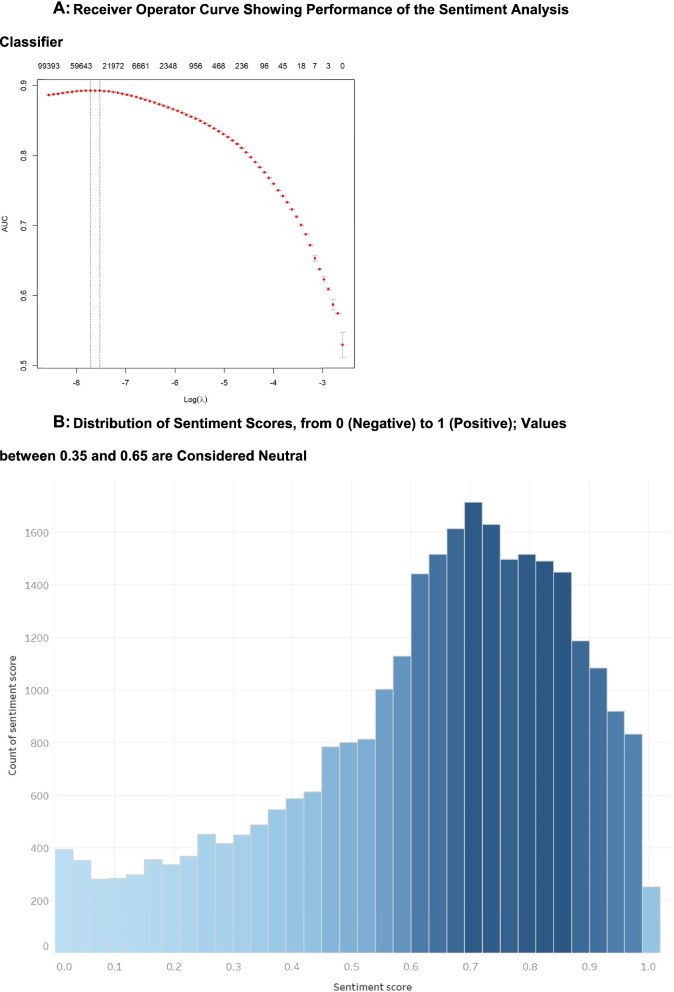
**A** Receiver operator curve showing performance of the sentiment analysis classifier **B** Distribution of sentiment scores, from 0 (negative) to 1 (positive); values between 0.35 and 0.65 are considered neutral

**Fig. 5:**
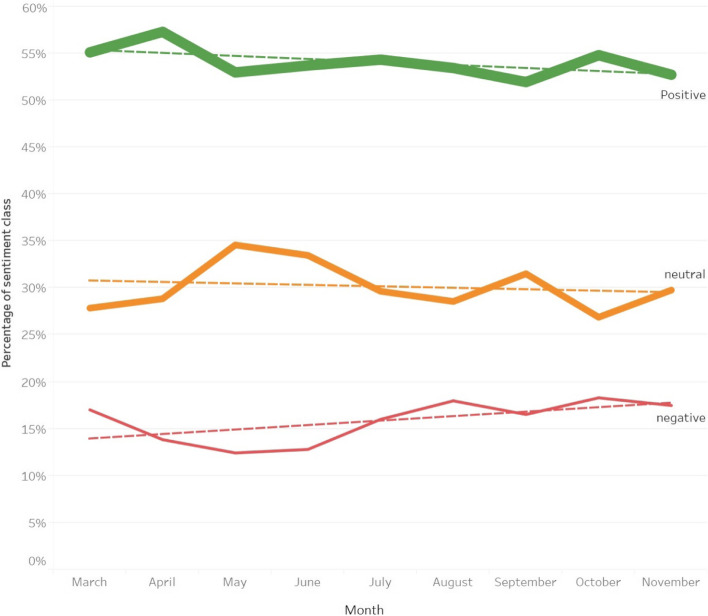
Changes in sentiment from March 03 to November 30, 2020 shown across monthly intervals

Our sentiment analysis algorithm using the GLMnet classifier to categorize the polarity of the tweet sentiments had an AUC of 0.894 as shown in Fig. 4A, which indicates a good ability for our classifier to distinguish between the different classes of negative and positive sentiments. Sentiments across all tweets for the 9-month period analysed were classified as positive (54.4%, *n* = 15 612), neutral (30.7%, *n* = 8803), and negative (15%, *n* = 4298), as shown in Fig. 4B. The relative proportions of positive, negative, and neutral sentiments expressed on a month-to-month basis remained largely constant across these 9 months, as shown in Fig. 5.

### Emotion analysis

**Fig. 6:**
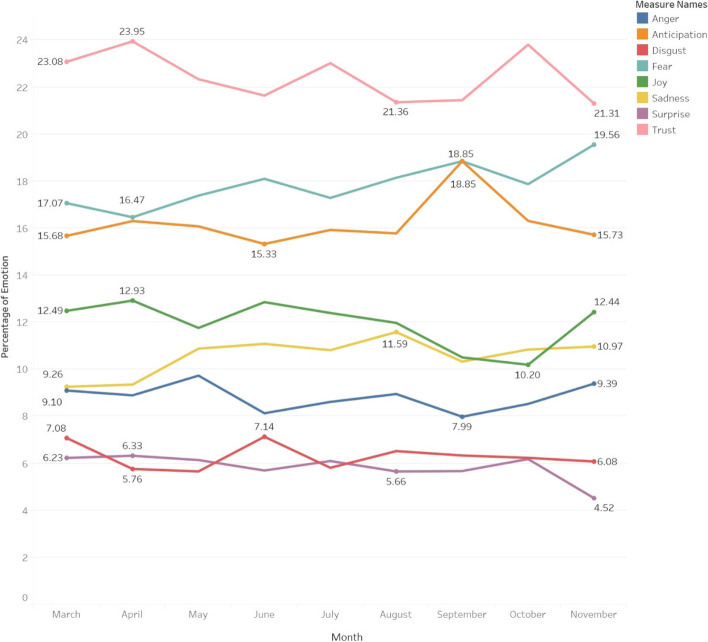
Changes in emotions represented from March 03 to November 30, 2020 shown across monthly intervals

When applying the algorithm employing the emotion lexicon to our tweet dataset, we were able to crosslink these emotions with text words within the tweets. The most prevalent emotion identified in the tweets was related to trust, which was associated with a total of 21,255 words. This was followed by fear (*n* = 16,410), anticipation (*n* = 15,080), joy (*n* = 11,407), and sadness (*n* = 9669). Anger (*n* = 8378), disgust (*n* = 5881), and surprise (*n* = 5621) were the least represented of the eight emotions in our dataset. The relative proportions of represented emotions expressed on a month-to-month basis remained largely constant across these 9 months, as shown in Fig. 6. It is important to note that the emotions are reflective of a word itself, and not a tweet. In Table 2, we provide illustrative examples of tweets classified as positive, neutral, and negative using sentiment analysis.

## Discussion

Over recent years, social media has become an increasingly popular generator and source of data that has interested a wide range of researchers [49]. The use of internet (including social media) data in studies, such as content and sentiment analyses, overcome some of the limitations of traditional social science research methods that rely on time-consuming, costly, retrospective, time-lagged, and small-scale approaches (e.g. surveys and interviews) [24, 50, 51]. In the context of pandemics, some research has even found that social media can be used to predict and detect one [52–54]. Further to this, once a pandemic has been identified, social media data can also be used to track public perceptions of the disease in question [22, 24, 55, 56]. One topic in the context of a pandemic, which has not been well-studied across social media, is the mention of CAIM. Yet, this topic is arguably of great interest given that a wide variety of CAIMs are being touted as preventative or curative against COVID-19 [57–59]. In fact, WHO Director General Tedros Adhanom Ghebreyesus at the Munich Security Conference on February 15, 2020 is quoted saying “We’re not just fighting an epidemic; we’re fighting an infodemic” in reference to rampant spread of misinformation, most notably across social media platforms [60].

In the present study, we conducted a sentiment and emotion analysis of Twitter data to explore what is said about CAIM in the context of COVID-19. To our knowledge, this is the first study to provide insights into the sentiments expressed by Twitter users at the intersection of CAIM and COVID-19. The majority of the tweets we identified and analyzed carried a generally positive sentiment. This was reflected in the emotional representation of "trust" with the highest word count in the dataset, an emotion that is frequently considered positive. We need to note the difference between the sentiment analysis of a tweet and the lexicon analysis using the Syuzhet package, as sentiment analysis is a whole tweet representation while the emotion lexicon is a word-based analysis. The latter algorithm compares words in the dataset to the NRC Sentiment and Emotional Lexicon, and it correlates words to eight standard emotions (anticipation, trust, joy, surprise, fear, sadness, anger, and disgust). From these patterns, the CAIM-related content being shared via Twitter would indicate support for CAIM interventions for COVID-19. This is in line with a plethora of published research studies that have found that the general public, across a number of different countries, tend to view CAIMs favourably and their usage continues to increase [61–65]. Over the course of our study, from March to November 2020, though the volume of tweets related to CAIM went down from the peak in May, the sentiments and emotions expressed in tweets were constant. From Table 1 and Fig. 2, as well as the illustrative tweets in Table 2, we see a focus on vitamins for prevention and treatment, which is also not entirely surprising given that across various surveys vitamins are known to be the most commonly used CAIMs [66, 67]. In fact, the 2012 National Health Interview Survey found that across all types of CAIM, natural health products (including vitamins) were the most commonly used among Americans [68].

**Table 1:** Top 10 Most frequent terms from the dataset of 28 714 CAIM-related COVID-19 Tweets

Term	Tweet count
vitamin	17 527
vitamins	3736
herbal	3577
Ayurveda	3281
herbs	2100
homeopathy	1355
traditional medicine	659
homeopathic	561
herbal medicine	556
naturopathy	238

**Table 2:** Illustrative examples of tweets with a positive, neutral, and negative sentiments

Tweet text	Sentiment score
If yall put MORE Trust in Herbal Remedies instead of ALL this #BigPharma Prescription Shit MAYBE you could see that the only ones who will PROFIT from this #COVID19 PLANdemic IS them and the Banks! https://t.co/63G6THEgTH	positive
Even in these troubled times, do not underestimate the benefits of a simply daily #walk. Choose your location carefully but take every opportunity you can to enjoy some fresh air, sunlight and vitamin D. Learn more on Sarahs Style & Dcor #blog #COVID19 https://t.co/KFx28wcasXhttps://t.co/63v73iMiOH	positive
Everything you need to know about #COVID19 but your government is too afraid to tell you. Get some sunshine on your skin. Eat vitamin D rich foods, and/or supplement. Wear a mask if somewhere crowded. https://t.co/AomlgyAeTC//t.co/https://t.co/xigTQ8SK5Ehttps://t.co/SdKvDBcV1Q	neutral
It still surprises me that there is not more media and doctors on television telling us to strengthen our immune system, take vitamins, eat healthily, get sunlight They only seem to be talking about vaccine and drugs that are in the distant future #COVID19 #coronavirusuk	neutral
A positive test doesn't mean a healthy person is going to be sick. Also it doesn't say for the sick persons if this virus is responsible for the illness. Fear creates diseases. Be cautious but not fearful. Boost your immune system get vitamin D3 or sunlight once a day #coronavirus	negative
Disgusting NHS in go-slow on Hydroxychloroquine trials to "justify" the #Lockdown. Delays will probably needlessly kill 100's of patients. Only 2 hospitals No Zinc No Z-Pak or other antibiotic No mention of Vitamins C or D https://t.co/yJunw9PFAE #COVID19 #Covid19	negative

### Comparative literature

To the authors’ knowledge, informed by preliminary searches of the academic literature, the present study is the first to conduct a sentiment and emotion analysis with the intent of understanding general CAIM content related to COVID-19 generated on Twitter. If we are to look outside of this intersection of topics, however, a growing number of studies involving social media data have been published relating to COVID-19. Some of these provide a more generalized overview of public COVID-19 discussions. Xue et al. [69] used unsupervised machine learning, qualitative analysis, and sentiment analysis to understand Twitter users’ discourse and psychological reactions to COVID-19, finding that while information relating to treatments and symptoms were not prevalent topics, fear of the unknown nature of the disease was dominant across all identified themes. Hung et al. [70] also applied machine learning methods to analyze data collected from Twitter including to identify the social network’s dominant topics and whether the tweets expressed positive, neutral, or negative sentiments. They identified 5 main themes including: health care environment, emotional support, business economy, social change, and psychological stress. Of approximately 900 000 tweets analyzed, their sentiment analysis classified 48% of tweets as having a positive sentiment, 21% as neutral, and 31% as negative. Abd-Alrazaq et al. [71] leveraged latent Dirichlet allocation (a type of NLP) for topic modelling to identify topics discussed in the tweets relating to the COVID-19 pandemic, in addition to conducting a sentiment analysis. They identified four main themes associated with their subset of included tweets including: origin of the virus; its sources; its impact on people, countries, and the economy; and ways of mitigating the risk of infection. They also found that the mean sentiment was positive for 10 topics and negative for 2 topics (first, COVID-19-caused deaths, and second, an increase in racism). Based on their findings, they noted that a more proactive and agile public health presence on social media is warranted to combat the spread of misinformation.

Other studies have focused their objectives on identifying types or prevalence of misinformation. Mackey et al. [72] used NLP and deep learning to detect and characterize illicit COVID-19 product sales using Twitter and Instagram data. They identified a few hundred tweets and posts, respectively, containing questionable immunity-boosting treatments or involving suspect testing kits, as well as a small number of posts about pharmaceuticals that had not been approved for COVID-19 treatment. Kouzy et al. [73] conducted searches on Twitter related to COVID-19, then summarized and assessed individual tweets for misinformation in comparison to verified and peer-reviewed resources, ultimately concluding that medical misinformation and unverifiable content were being propagated at an alarming rate. In contrast, Singh et al. [74] also analysed COVID-19-related Twitter content but found that while discussions surrounding myths and links to poor quality information did exist, their presence was less dominant than other crisis-specific themes. Krawchuk et al. [75] conducted a descriptive study which detailed Twitter activity regarding spinal manipulative therapy and claims made that it increases or boosts immunity. They found that misinformation linking spinal manipulation and increased immunity increased dramatically at the onset of the COVID-19 crisis. Lastly, Yang et al. [76] sought to understand the landscape and propagation of COVID-19 misinformation and its correction on Sina Weibo, China’s largest microblogging website. While the authors did not specifically aim to capture CAIM-related information, they found that rumours surrounding false or untested therapies/measures (e.g., traditional Chinese medicine, saline water, firecrackers, and even smoking) prevention and treatment of COVID-19 were among the topics commonly circulated.

### Addressing COVID-19 and CAIM misinformation on social media

Misinformation has been defined as “false and inaccurate information that is spread intentionally and unintentionally” [77] and is known to spread on social media networks easily and quickly [78, 79]. Due to the negative potential influence on people’s health practices, health misinformation has received more scholarly attention, especially since the beginning of the COVID-19 pandemic [80–82]. It is particularly harmful because: 1) people are more likely to trust the information after they have been exposed to it, 2) correcting misinformation is time-consuming and resource-intensive, and 3) even after correction, it may continue to influence attitudes and behaviours, reflecting a phenomenon known as “belief echoes” [83, 84]. Correcting disinformation has become more complex and difficult as social media platforms have grown in popularity, catalysing the quick and widespread spread of misinformation. Social media networks are highly afflicted by misinformation, and it is a challenge to block or flag (re)transmission due to a lack of professional gatekeeping [85, 86]. This issue is compounded by the fact that health information seeking and scanning behaviours on social media networks increase when faced with a public health crisis [87], with COVID-19 being no exception [88, 89].

The WHO provides seven items for individuals to identify misinformation, as follows: 1) assess the source, 2) go behind the headlines, 3) identify the author, 4) check the date, 5) examine the supporting evidence, 6) check your biases, and 7) turn to fact-checkers [90]. The WHO has also published a webpage with weblinks to report misinformation found on commonly used social media platforms [91]. On a global scale, the WHO and its partners are leading three initiatives to combat misinformation online. The first involves changing social media policy and guidelines, by working with content providers such as YouTube, to reduce and remove videos containing misinformation. The second involves reporting misinformation, whereby several social media platforms granted the WHO access to fast-track reporting systems, allowing for quicker tagging and removal of content containing misinformation. Lastly, the WHO has leveraged data insights by working with YouTube, Google, and Facebook, among others, to understand where misinformation is most rampant, to target the delivery of science-based health information where it is most needed [92]. Specific to CAIM misinformation, the NCCIH offers an online resource known as “Know the Science” which provides interactive modules allowing users to learn about topics such as making sense of health research and deciding whether health news stories contain missing, misleading, or conflicting information, along with other information-related resources offered by the US National Institutes of Health and the US Centers for Disease Control and Prevention [93].

### Future directions

Several future directions could be followed, based on the present study as well as emerging research in this topic area. As misinformation surrounding the COVID-19 pandemic is both rampant and pervasive on Twitter, among other social media platforms, several researchers have begun developing tools to track such misinformation. Sharma et al. [94] designed a dashboard to track misinformation on Twitter, which aims to identify false, misleading, and clickbait contents from collected tweets. Al-Rakhami et al. [95] has proposed an ensemble-learning-based framework for verifying the credibility of a vast number of tweets, which classifies tweet information based on tweet- and user-level features into two categories, either “credible” or “non-credible”. Tools such as these can be applied to Twitter datasets containing information at the intersection of CAIM and COVID-19 to both compare with and validate our findings. Additionally, while our sentiment and emotion analysis provides us with insight into the polarity of sentiment and the emotions expressed in our dataset, a qualitative content analysis could identify: specific themes pertaining to this intersection of topics, trending topics, ideas most commonly linked in the text, and characterize who is generating and sharing related tweets.

### Strengths and limitations

We extracted a large number of tweets that were posted over the first 9 months of the COVID-19 pandemic between March 03, 2020, and November 30, 2020 inclusive and applied two different methods to analyze the tweet dataset. We employed a supervised machine learning approach utilizing the Text2Vec package for our sentiment analysis. The purpose of this method was to acquire generalizable results built on labelled data which provided results for each tweet as a whole based on the combination of words (respecting their locality and relation to each other), rather than a lexicon-based analysis which treats each word as a separate entity. Using the highly cited Sentiment140 dataset for training our sentiment analysis model is a strength as the dataset contains 1.6 million machine labelled tweets categorized by polarity. Finally, the Syuzhet package in R is considered a good machine learning technique to provide an emotion representation of the words within the tweets based on the NRC emotion lexicon database. We applied considerable rigour in developing our search strategy by consulting reviews of CAIM, MeSH terms, and conducting trial searches within Twitter to ensure that we identified the most relevant and used terms. It is also worth noting that few sentiment analyses published to date have analyzed or compared sentiments over multiple time periods. As opposed to capturing all tweets posted on one day or a series of days, unique to our study is the fact that we captured tweets across a period of 9 months which allowed us to compare trends over time as the pandemic progressed.

Limitations include the fact that we did not account for all CAIMs, as they represent a dynamic and wide range of therapies. This was mitigated by the preliminary searches of Twitter for the CAIMs most commonly mentioned in tweets that informed our decision on what terms to include. A further limitation is that sentiment has been classified along the continuum of positive to negative, without additional approaches to detect such linguistic elements as sarcasm, context, and complex emotions or sentiment, which are evident in the tweets illustrated in Table 2 [96]. The reliability of a model relates to its consistent performance throughout the period and conditions it is being tested [97]. Our model achieves an AUC score of 0.89 which is considered good performance for a classifier. The reliability of sentiment analysis models can be variable, with differences among them [97, 98]. We aimed to mitigate this concern by using Sentiment140 [29], a large dataset of 2.5 million labelled tweets which has been used in several other sentiment analysis studies in the context of health research [99–101]. Using this to train our model boosts confidence in its performance and reliability. During the initial phases of the study we relied on the Twitter rest/standard API, which does not allow a tweet retrieval past a certain time. Due to this limitation within the Twitter API, we relied on the Harvard Dataverse COVID-19 dataset, which had not been updated past December 03, 2020 at the time we conducted our analysis. As such, we have a narrow window of time reflected in the analyzed tweets. Given that this dataset has since been updated, in the future, we could apply our methods to discern how the sentiments and emotions in tweets have evolved as the pandemic has progressed. We limited our tweets to originals and in English. Given the global nature of the pandemic and the regional differences in CAIM treatments, we likely have missed relevant tweets. Future research on the amplification of messaging via retweets could also lead to new insights into the spread of CAIM-related content in the context of this pandemic.

## Conclusions

We conducted a sentiment analysis with the objective of understanding what was being mentioned about CAIM in the context of the COVID-19 pandemic on Twitter. A total of 28 713 English-language tweets were analyzed. The most common CAIM-related hashtag used was #vitamin followed by #ayurveda. Most of the tweets were classified as positive (54%), followed by neutral (31%) and negative (15%). The most frequent emotions expressed across tweets was trust, followed by fear. Social media continues to be an important source of data that provides a range of advantages over traditional data sampling techniques, such as surveys and interviews. The use of sentiment analysis on Twitter data at the intersection of CAIM and COVID-19 provides insight into how such data is being disseminated. Our findings warrant further qualitative investigation of the emotions identified across tweets analysed, which could be used to combat the spread of misinformation and inform improved public health strategies surrounding the use of social media information.

## Data Availability

All relevant data are included in this manuscript.

## Abbreviations

API: Application programming interface
AUC: Area under the ROC curve
CAIM: Complementary, alternative, and integrative medicine
COVID-19: Coronavirus disease 2019
DTM: Document term matrix
NCCIH: National Centre for Complementary and Integrative Health
NLP: Natural language processing
NRC: National Research Council Canada
ROC: Receiver operator characteristic
SARS-CoV-2: Severe acute respiratory syndrome coronavirus 2
WHO: World Health Organization
